# First Identification and Genomic Characterization of Mammalian Orthoreovirus Type 1 in a Heavily Polluted Stream from an Underserved Urban Settlement in Brazil

**DOI:** 10.3390/v18101161

**Published:** 2026-10-19

**Authors:** Anderson Vicente de Paula, Layla Honorato, Lucy Santos Vilas Boas, Anna Sara Shafferman Levin, Maria Tereza Pepe Razzolini, Ronalda Silva de Araújo, Mikaela Renata Funada Barbosa, Antonio Charlys da Costa, Maria Cassia Mendes-Correa

**Affiliations:** 1 Laboratory of Medical Investigation in Virology (LIM-52), Instituto de Medicina Tropical de São Paulo, Faculdade de Medicina, Universidade de São Paulo, Av. Dr. Enéas de Carvalho Aguiar, 470, São Paulo 05403-000, Brazil; anddeblair@yahoo.com.br (A.V.d.P.); lucyvilas@yahoo.com.br (L.S.V.B.); charlysbr@yahoo.com.br (A.C.d.C.); maria.cassia@hc.fm.usp.br (M.C.M.-C.); 2 Department of Infectology and Tropical Medicine, Faculdade de Medicina, Universidade de São Paulo, São Paulo 01246-903, Brazil; gcih.adm@hc.fm.usp.br; 3 Department of Environmental Health, Faculdade de Saúde Pública, Universidade de São Paulo, São Paulo 01246-904, Brazil; razzolini@usp.br; 4 NARA—Center for Research in Environmental Risk Assessment, São Paulo 01246-904, Brazil; 5 Department of Environmental Analysis, Division of Microbiology and Parasitology, Environmental Company of the State of São Paulo (CETESB), São Paulo 05459-900, Brazil; rsaraujo@sp.gov.br (R.S.d.A.); mrbarbosa@sp.gov.br (M.R.F.B.)

**Keywords:** reovirus, mammalian orthoreovirus, reassortant, virus replication, transmission, urban settlement

## Abstract

Mammalian orthoreovirus (MRV), a member of the family *Spinareoviridae* within the order Reovirales, has a ten-segmented double-stranded RNA genome and is known for its environmental stability and broad host range, including humans. Although human MRV infections are usually asymptomatic or mild, occasional severe MRV-associated disease has been reported. Despite the widespread presence of MRV, particularly in contaminated water, genomic data from South America are limited, especially from environmental sources in underserved urban settlements. In this study, we isolated a virus from a heavily polluted stream running through an underprivileged, underserved, urban settlement in São Paulo, Brazil. The causative agent was identified as a novel MRV strain (MRV1_SP/BR) by whole-genome Illumina sequencing, and its presence was subsequently confirmed by RT-qPCR. This analysis revealed a complex mosaic genome architecture indicative of genetic reassortment. Based on phylogenetic analysis of the S1 segment, the strain was genetically classified as MRV-1, whereas its remaining segments showed high nucleotide identity to sequences previously detected in humans and bats. These findings provide the first genomic characterization of a reassortant MRV detected in an urban stream in South America and expand the available information on MRV diversity in sewage-impacted aquatic environments.

## 1. Introduction

Mammalian orthoreoviruses (MRVs) are double-stranded RNA viruses belonging to the genus *Orthoreovirus*, family Spinareoviridae, and order Reovirales [1]. Their genome comprises ten segments (L1–L3, M1–M3, and S1–S4), which allows frequent genetic reassortment and contributes to their broad host range, infecting various mammalian species, including humans [1]. Currently, four MRV genotypes are recognized, defined by the phylogenetic classification of the S1 segment [2].

Human MRV infections are widely distributed and are typically asymptomatic or mild, sometimes presenting with transient respiratory or enteric symptoms [3,4,5]. However, an increasing number of sporadic cases involving acute respiratory infections, hemorrhagic gastroenteritis, encephalitis, and systemic illness have increasingly been reported in diverse age groups and immune statuses, highlighting their emerging relevance as human pathogens [6,7,8,9,10]. Additionally, novel reoviruses from animals have been identified, some of which have crossed the species barrier and infected humans, raising concerns about their zoonotic potential [6,10,11]. Novel reassortant MRV strains of these viruses have also been detected in travellers, which raises concern about the adaptability and transmission potential of MRVs [12,13].

Notably, the robust structure of MRVs plays a crucial role in their transmission and persistence. As non-enveloped viruses with a double-layered capsid, they exhibit exceptional stability, allowing them to resist physical and chemical inactivation [11]. This resilience facilitates not only direct person-to-person transmission but also indirect infection through water, food, or surfaces, enhancing their persistence and circulation across multiple hosts and ecosystems [11,14,15,16,17]. Consequently, MRVs are among the most common enteric viruses found in wastewater and are increasingly being studied as indicators of faecal pollution in watersheds, coastal areas, and groundwater sites [11,16,17,18]. This combination of environmental persistence and efficient indirect transmission likely explains the high seroprevalence already observed in early childhood [5,19].

Despite their widespread presence in the environment, publicly available MRV genomic data remain limited. Although the characterization of MRVs in untreated water near densely populated areas is essential for understanding their diversity, evolution, and potential impact on public health, studies in this area remain scarce. In underserved urban settlements (*favelas*), where there is a lack of sanitary infrastructure, the persistence of MRVs increases the risk of human exposure. Additionally, the mixing of human and animal excreta in these open settings may introduce viruses from multiple sources into aquatic environments, making these settings relevant targets for environmental surveillance.

In this study, we present the first genomic characterization of an MRV strain from a heavily polluted stream in an underserved populated favela in São Paulo, Brazil, providing insights into its genomic composition, phylogenetic relationships, and reassortment history.

## 2. Materials and Methods

### 2.1. Sample Collection and Processing

**Figure 1:**
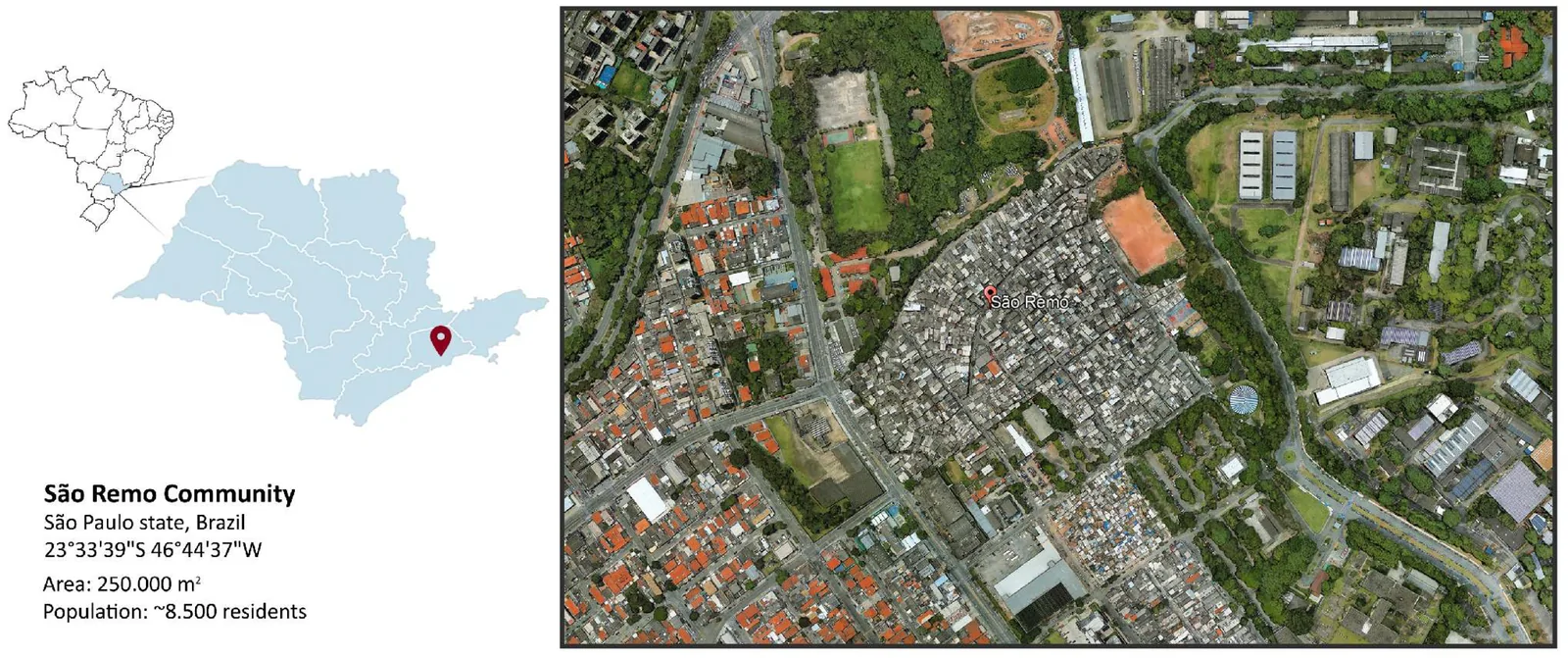
Location of the São Remo Community in the city and state of São Paulo, Brazil.

A total of 31 stream water samples (1 L each) were collected weekly from the Riacho Doce stream, a heavily polluted watercourse that flows through the São Remo community, an underserved area in the western region of São Paulo, Brazil. This region, with an estimated population of approximately 8000 inhabitants, lacks a sewage system, and all domestic effluents are discharged directly into the stream (Figure 1). The samples were originally collected between May and November 2020 as part of a previous study aimed at evaluating the presence of SARS-CoV-2 in the stream and its correlation with COVID-19 cases in the community [20].

The samples were collected according to the *Standard Methods for the Examination of Water and Wastewater* [21]. All samples were transported in refrigerated containers (0 °C to 8 °C) to the Division of Microbiology and Parasitology at CETESB (*Environmental Company of the State of São Paulo)* and processed within 24 h. For viral concentration, 40 mL of each sample were subjected to ultracentrifugation at 110,000× *g* for 1 h at 4 °C. The resulting pellet was resuspended in 4 mL of 0.25 N glycine buffer (pH 9.5) and incubated on ice for 30 min. After centrifugation at 3000× *g* for 20 min to remove suspended solids, the supernatant was ultracentrifuged again under the same conditions. The final viral pellet was resuspended in 0.3 mL of PBS and stored at −80 °C. These samples were then sent to the Virology Laboratory LIM-52 at the Institute of Tropical Medicine, University of São Paulo.

### 2.2. Viral Isolation

Because virus isolation was conducted within the scope of a previous study focused on the detection of SARS-CoV-2 in the stream, only the seven samples previously confirmed positive for SARS-CoV-2 by two independent RT-qPCR assays (targeting N1 and N2 genes at CETESB, and E and S genes at the Virology Laboratory LIM-52) were selected for virus isolation in Vero CCL-81 cells (ATCC^®^ CCL-81™, Manassas, VA, USA). Cells were seeded in 25 cm^2^ polystyrene culture flasks (Jet Biofil^®^, Guangzhou, China) at a density of 5 × 10^5^ cells/mL in DMEM supplemented with 5% heat-inactivated fetal bovine serum (Vitrocell Embriolife, Campinas, Brazil) and incubated overnight at 37 °C in a 5% CO_2_ atmosphere. On the following day, the supernatant was removed, and the viral concentrate was added to the cell monolayer. After 1 h of incubation, the medium was replaced with DMEM containing 2.5% fetal bovine serum and 1% penicillin–streptomycin. Cultures were maintained at 37 °C in 5% CO_2_ and monitored daily for cytopathic effects (CPE) for at least five days, with incubation continuing for a total period of at least two weeks. A minimum of two subcultures (passages) were performed for each sample. RT-PCR was conducted on supernatants collected after the second passage to assess the presence of SARS-CoV-2.

Cytopathic effects were observed in two of the seven inoculated cultures during the first passage. Although these samples had initially tested positive for SARS-CoV-2, subsequent RT-PCR assays performed on supernatants from the following passage were negative for SARS-CoV-2, suggesting that the observed CPEs were induced by another viral agent. To elucidate the etiology, the samples were subjected to molecular testing for a comprehensive panel of other viral pathogens, including enteroviruses, varicella-zoster virus, dengue virus, chikungunya virus, yellow fever virus, parechovirus, human herpesviruses 5, 6, and 8, herpes simplex viruses 1 and 2, and adenovirus. All results were negative, thereby excluding these agents as potential causes.

### 2.3. Sequencing and Bioinformatics Analysis

To identify the causative agent of the cytopathic effect, sequence-independent amplification of viral RNA was performed. Library preparation and sequencing were performed according to the protocol described by Ferreira et al., (2025), with minor modifications [22]. Briefly, 2 ng of double-stranded DNA generated from five microliters of total nucleic acid was processed using the Nextera XT Kit (Illumina, San Diego, CA, USA), following the manufacturer’s instructions. Sequencing was performed on the Illumina NovaSeq 6000 platform (Illumina, San Diego, CA, USA) using a 500-cycle paired-end strategy.

Raw paired-end sequencing reads were initially assessed using FastQC version 0.12.1 and processed with fastp version 1.2.0 to remove adapter sequences, low-quality bases, and short reads. The quality-filtered reads were assembled de novo using SPAdes version 4.2.0 in metagenomic mode (metaSPAdes). The resulting contigs were screened using MMseqs2 version 18-8cc5c, and candidate viral contigs were subsequently confirmed by BLASTn searches against the NCBI nucleotide database. Contigs corresponding to all ten mammalian orthoreovirus genome segments were identified. Quality-filtered reads were then mapped back to the assembled MRV segment contigs using BWA-MEM version 0.7.19. Alignment files were converted, sorted, and indexed using SAMtools version 1.23, which was also used to calculate segment-specific read counts and mean coverage depth. These sequencing metrics are provided in Supplementary Table S1. Sequence variants were identified using BCFtools version 1.23, and final consensus sequences were generated after applying predefined minimum base-quality and read-depth thresholds.

Nucleotide sequences were evaluated using BLASTn version 2.15.0 to determine similarity with known viruses deposited in the NCBI GenBank database. Closely related MRV genomes and prototype strains were retrieved and aligned using MAFFT version 7.4 in Unipro UGENE v52.0. Phylogenetic analysis was conducted using the maximum likelihood method with the GTR+Gamma model and 1000 bootstrap replicates in IQ-TREE version 3.1.0. Nucleotide identity analysis was performed in MEGA X, and potential reassortment events among genome segments were assessed using concatenated whole-genome alignments and similarity plots generated with SimPlot++ version 1.3. The complete genome sequence of the São Remo MRV strain, designated MRV1_SP/BR, was deposited in GenBank under accession numbers PV209793 to PV209802.

### 2.4. Molecular Confirmation of MRV by RT-qPCR

To confirm the presence of MRV in the samples showing CPE, RNA was extracted using the QIAamp Viral RNA Kit (QIAGEN, Hilden, Germany), according to the manufacturer’s instructions. A one-step RT-qPCR assay was developed targeting a conserved region of the L1 genomic segment, which encodes the λ3 protein. Primers and probe were designed based on the MRV1_SP/BR sequence obtained by whole-genome sequencing and subsequently evaluated against MRV sequences available in GenBank to identify a conserved region shared among MRV genotypes. The following primers were used: forward primer TCACAATCCCCCATCACTATT, reverse primer CATCCATTTCTGCCAGTTCTT, and probe FAM ATAAATGGTATCAGGGCGCRCAGGAG-MGB. The reactions were prepared in 20 μL volumes using TaqMan Fast Virus 1-Step Master Mix (Thermo Fisher Scientific, Waltham, MA, USA), with final primer concentrations of 450 nM, probe at 125 nM, and approximately 100 ng of template RNA. The assays were performed in replicate using both the virus isolate obtained in cell culture and the corresponding original wastewater sample (primary sample). Two negative controls were included: nuclease-free ultrapure water (Thermo Fisher Scientific, Waltham, MA, USA) and a no-template control (NTC). The thermocycling protocol consisted of reverse transcription at 50 °C for 5 min, initial denaturation at 95 °C for 20 s, followed by 40 amplification cycles of 95 °C for 3 s and 60 °C for 30 s, performed on a QuantStudio 5 instrument. Data analysis was conducted using QuantStudio Design & Analysis Software v1.4.1 (Thermo Fisher Scientific, Waltham, MA, USA). Samples with a cycle threshold (Ct) value ≤ 38 were considered positive. The presence of MRV was confirmed in both CPE-positive samples.

## 3. Results

**Figure 2:**
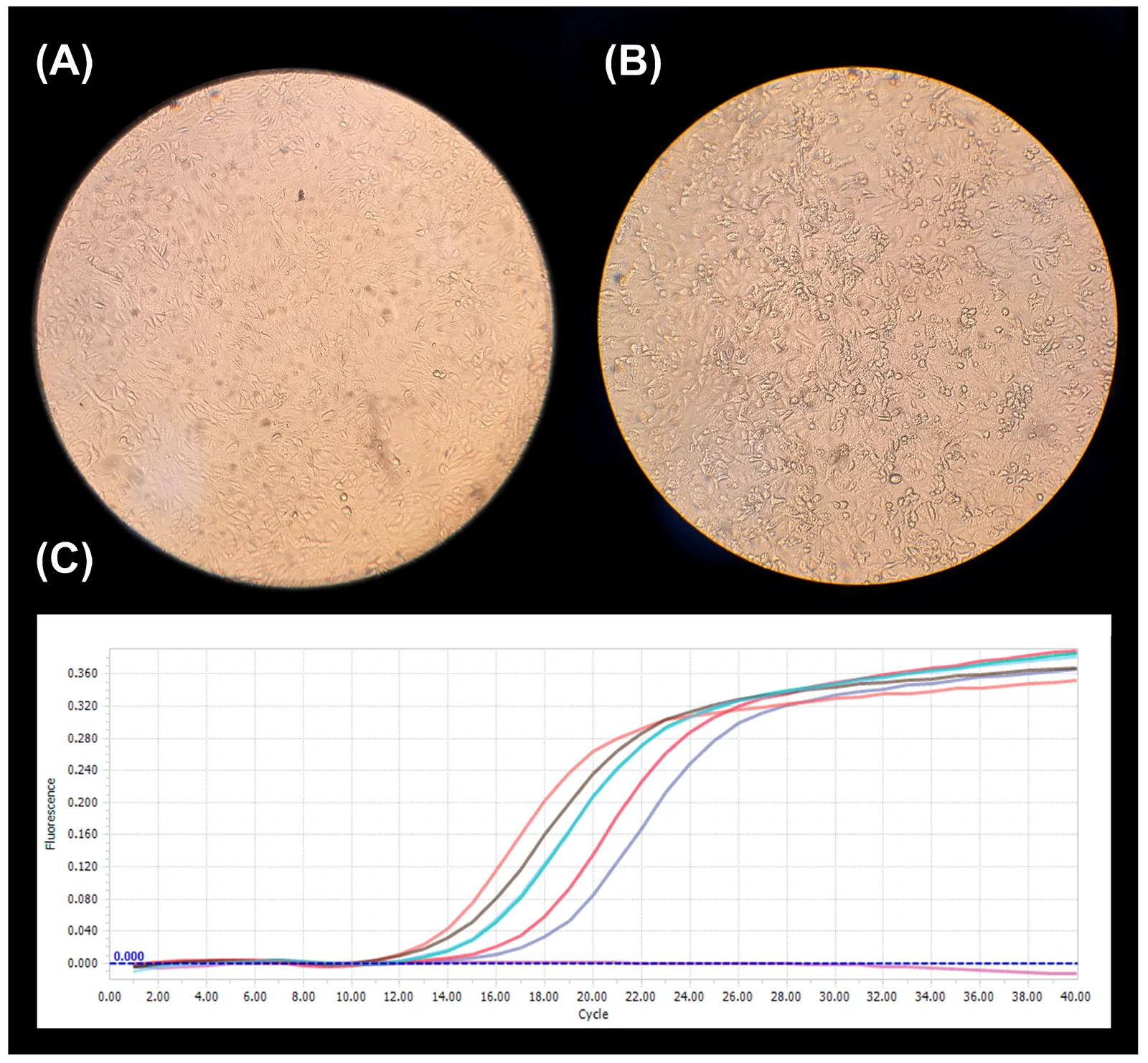
Cytopathic effect (CPE) and reverse transcription quantitative polymerase chain reaction (RT-qPCR) detection of the mammalian orthoreovirus (MRV) strain MRV1_SP/BR in Vero CCL-81 cells. (**A**) Uninfected control cells showing a confluent monolayer with preserved cell morphology. (**B**) Infected cells at 72 h post-infection showing cell rounding and disruption of the monolayer, consistent with CPE. (**C**) RT-qPCR amplification curves targeting the MRV L1 genomic segment in samples from five successive cell culture passages and a negative control, obtained using the QuantStudio 5 Real-Time PCR System (Thermo Fisher Scientific, Waltham, MA, USA). The horizontal axis indicates the amplification cycle number, and the vertical axis indicates fluorescence intensity.

**Figure 3:**
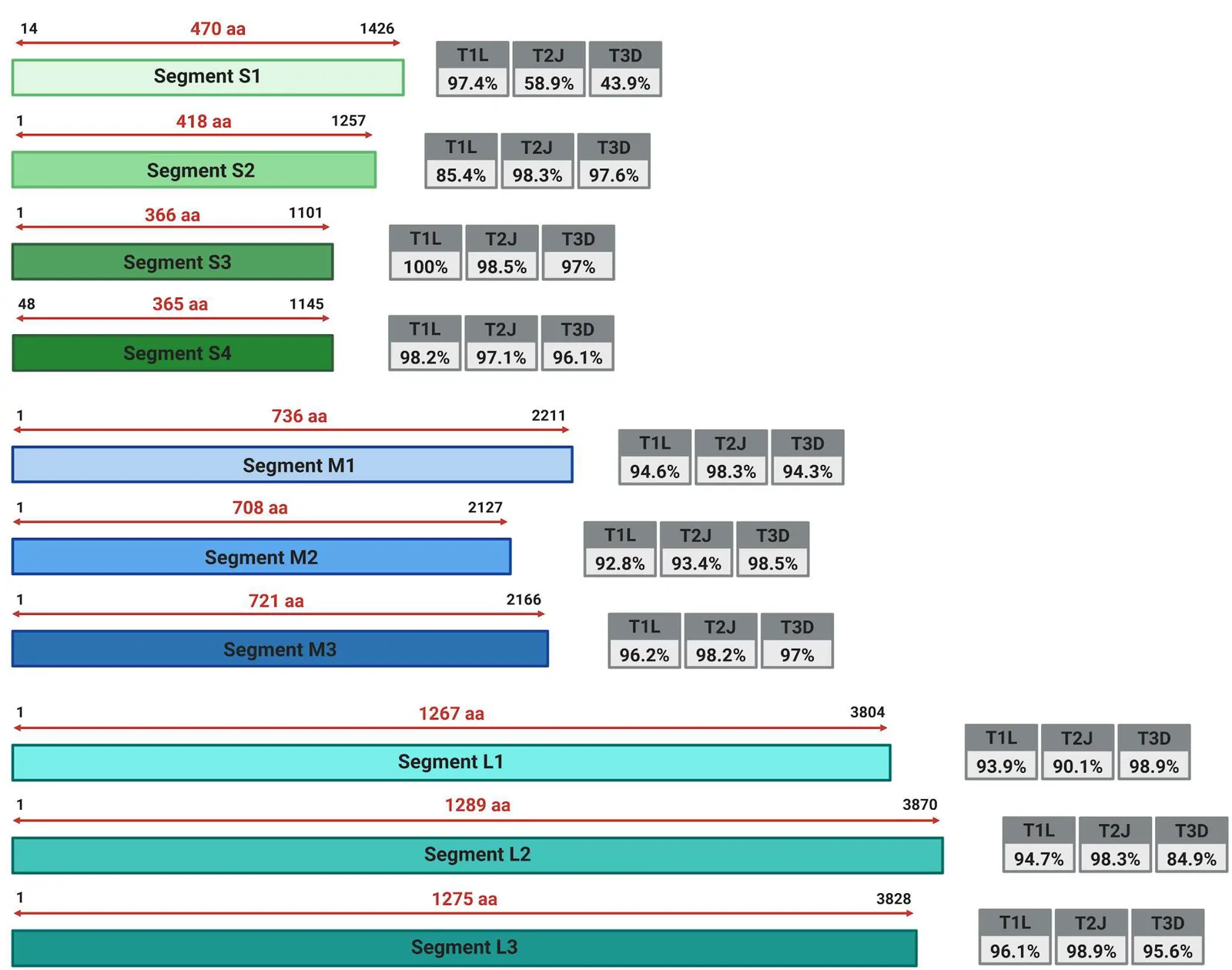
Schematic representation of the segmented genome of the Brazilian São Remo MRV1_SP/BR strain. Colored rectangles depict the coding regions of each genomic segment. The numbers flanking each segment indicate the start and end positions of the coding region. The numbers above the double-headed arrows represent the length of the coding region in amino acids (aa). The gray boxes on the right side of each segment display the nucleotide identity of MRV1_SP/BR compared with the reference strains MRV-1 Lang (T1L), MRV-2 Jones (T2J), and MRV-3 Dearing (T3D).

A cytopathic effect was observed in two of the seven inoculated cell cultures (Figure 2). Subsequent sequencing analysis recovered the complete genome of the novel Brazilian strain, named São Remo MRV1_SP/BR, encompassing all ten genomic segments. A total of 27,120 reads mapped to the MRV1_SP/BR genome. Segment-specific mean coverage ranged from 180× for S3 to 3710× for L1, while the recovered segment lengths ranged from 1101 to 3963 nt (Table S1). To assess its genetic relatedness, pairwise nucleotide identity comparisons were performed between MRV1_SP/BR and the three prototype mammalian orthoreovirus strains: MRV-1 Lang (T1L), MRV-2 Jones (T2J), and MRV-3 Dearing (T3D) (Figure 3).

The analysis revealed that the S1 segment exhibited 97.4% nucleotide similarity with a wastewater isolate from Canada (OR074680). Notably, apart from segment L1, which displayed a high nucleotide identity (98.9%) with a human-derived strain from Switzerland (KX932029), all other segments showed the highest nucleotide identity with bat-derived isolates from different species and geographic locations (Table 1). The nucleotide identity profile varied among genome segments. The L1, S1, S3, and S4 segments were most closely related to the corresponding segments of strains classified as MRV-1; the M2 segment was most closely related to a strain classified as MRV-3; and the L2, L3, M1, M3, and S2 segments were most closely related to strains classified as MRV-2.

**Figure 4:**
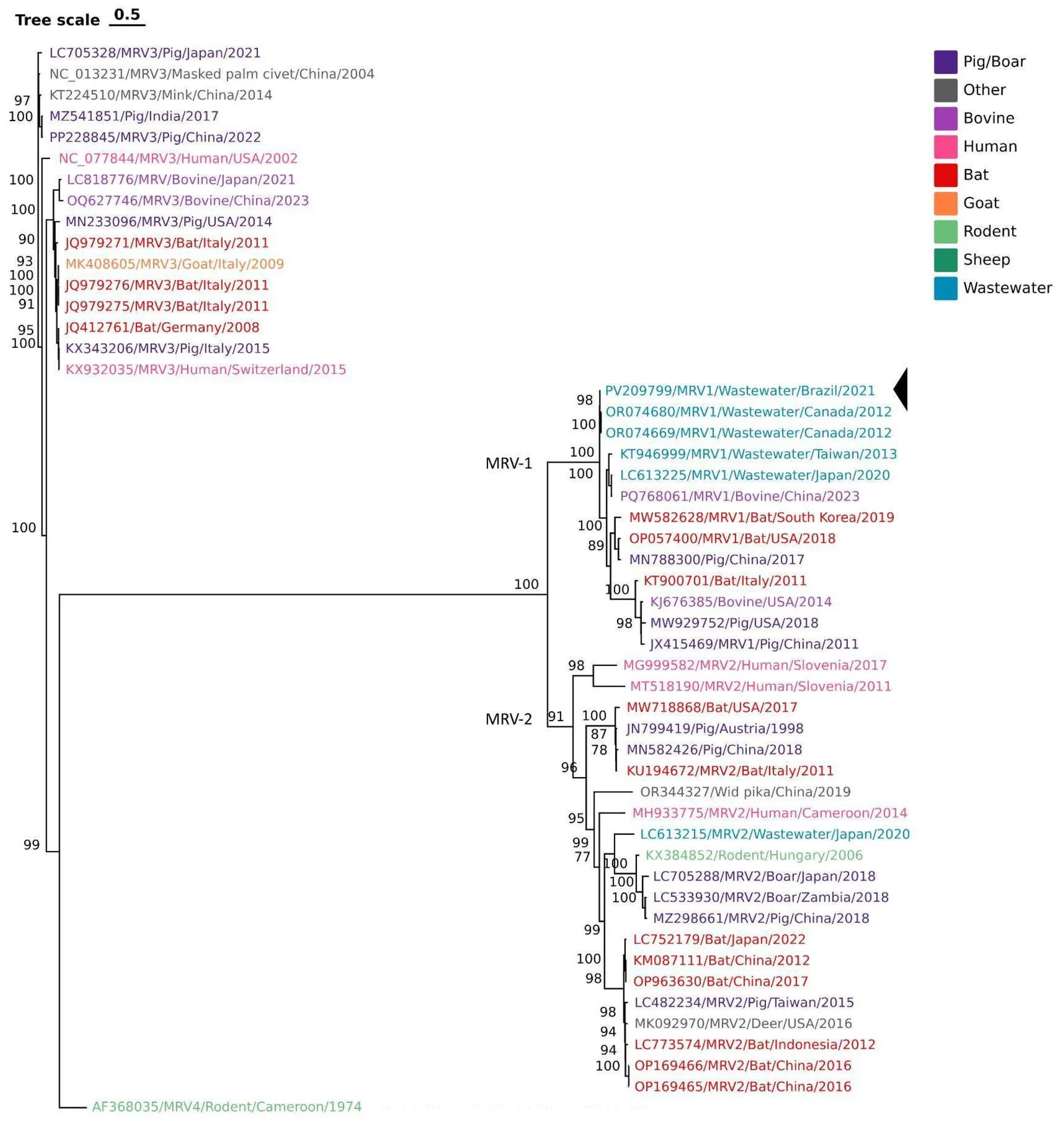
Phylogenetic tree of the complete S1 segment of the Brazilian São Remo MRV1_SP/BR strain, indicated by a black arrow, with representatives of the four S1-based genetic types. Different hosts/sources are highlighted in different colors. Accession number, host species, country, and year are indicated for each strain when available. The tree was inferred using the maximum-likelihood method under the GTR+G model, with 1000 bootstrap replicates.

Since nucleotide identity alone does not resolve evolutionary relationships, a detailed phylogenetic analysis was conducted using a maximum likelihood approach to infer the evolutionary clustering of each segment (Figures S1–S3). Phylogenetic analysis of the S1 segment placed MRV1_SP/BR within the MRV-1 clade, together with wastewater-derived strains from Canada (OR074680 and OR074669), supporting its genetic classification as MRV-1 (Figure 4).

**Figure 5:**
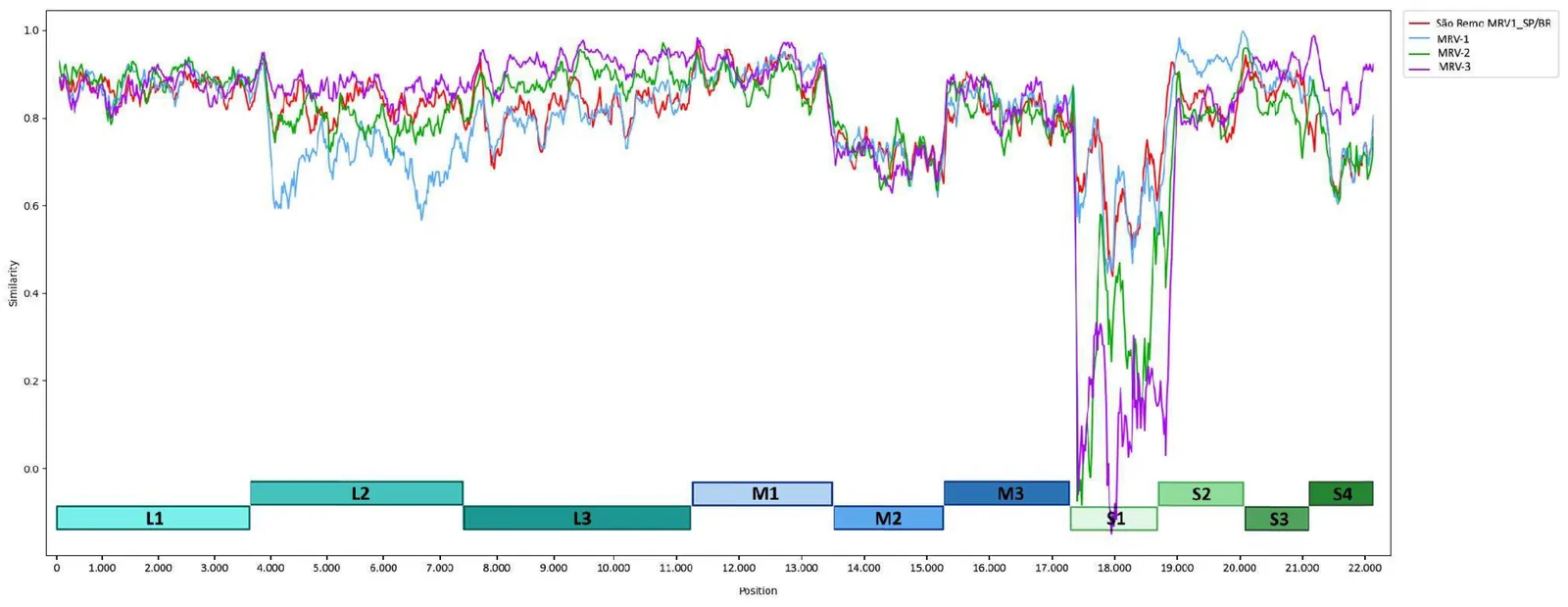
Similarity plot analysis of the whole-genome nucleotide sequence of the Brazilian São Remo MRV1_SP/BR strain compared with the most closely related complete MRV strains available in GenBank. The analysis was performed using the Jukes–Cantor distance. The colored curves represent São Remo MRV1_SP/BR (red), MRV-1 (light blue), MRV-2 (green), and MRV-3 (purple), as indicated in the plot legend. The colored blocks below the plot indicate the positions of the ten genome segments in the concatenated alignment: L1–L3 (shades of turquoise), M1–M3 (shades of blue), and S1–S4 (shades of green).

The phylogenetic analyses of the remaining segments revealed a complex pattern of topological incongruence, consistent with reassortment involving viral lineages previously detected in different host species. Segments L1, M2, M3, S2, S3, and S4 clustered with bat-derived isolates. In contrast, L2 and L3 clustered with a more diverse range of hosts, including pigs, sheep, and bats. The SimPlot analysis indicated possible intertypic genome reassortments. Most indications of a reassortment event were found for the L2 and S2 segments, for which São Remo MRV1_SP/BR exhibited higher nucleotide similarity to MRV-2 reference strains than to MRV-1 reference strains, suggesting that these segments may have originated from an MRV-2-like lineage. Additionally, while reassortment signals were evident in these genomic regions, other segments such as L1 and M2 displayed a high degree of conservation among strains representing different MRV genetic types (Figure 5).

Lastly, comparison with the previously reported Brazilian strain TO-151/BR, detected in a child with diarrhea in Tocantins, northern Brazil, revealed segment-specific nucleotide identities ranging from 47.0% for S1 to 94.5% for L3 (Table 2). The marked divergence of the S1 segment is consistent with the S1-based genetic classification of TO-151/BR as MRV-3 and MRV1_SP/BR as MRV-1.

## 4. Discussion

This study represents the first molecular characterization of a novel MRV isolate from an open, sewage-contaminated stream in Brazil. Whole-genome analysis revealed a mosaic architecture, with none of the ten genomic segments clustering consistently with a single reference strain. Instead, the segments exhibited distinct phylogenetic affinities, including an L1 segment most closely related to a sequence previously detected in a human sample and several segments most closely related to sequences previously detected in bats (L2, L3, M1, M2, M3, S2, S3, and S4). These relationships support the reassortant nature of MRV1_SP/BR but do not establish the host origin of the individual segments.

Reassortment is a key mechanism in the emergence of new reovirus variants [13,23,24,25]. Due to their segmented nature, co-infection by multiple MRV lineages may generate novel strains with potentially increased virulence or transmission efficiency. Previous studies have reported reassortment events between MRV strains [12,13,17,23,25,26,27,28], and similar mosaic genomes have been documented, combining genes from bats, livestock, and humans [10,17,28,29,30,31]. For instance, Ouattara et al. described an MRV from a child with necrotizing encephalitis whose genome was a reassortant of human and porcine strains [9]. Steyer et al. described an MRV from a child with acute gastroenteritis that shared eight of its ten segments with German bat isolates [10]. These previous reports illustrate that MRV reassortment may involve lineages detected in different hosts; however, the host-associated sequence similarities observed in the present study do not by themselves demonstrate cross-species transmission.

Bats host a wide diversity of orthoreoviruses. Cross-species transmission from bats to humans has been demonstrated for pteropine orthoreoviruses [32,33,34,35]. MRV transmission is not restricted to direct contact and also occurs through indirect environmental contamination, with reovirus particles detected in various environmental samples [10,11,15,17,36]. Their persistent stability in the environment suggests that indirect transmission via contaminated food, water, or surfaces may play a significant role in viral spread [11,14,17].

Conditions such as those in São Remo, characterized by inadequate sanitation, high population density, and frequent exposure to open, contaminated water, may increase human exposure to environmentally persistent viruses. Similar conditions are found in underserved settlements worldwide, where approximately 3.5 billion people still lack access to basic sanitation [37]. Although MRVs are generally considered nonpathogenic, seroprevalence rates can reach up to 50% in early childhood, with the virus often causing mild respiratory and enteric symptoms that frequently go undocumented [19,38]. Rare severe MRV-associated outcomes, including meningitis, encephalitis or necrotizing encephalopathy, and severe gastroenteritis, have been reported [5,8,9,10,39,40]. These reports highlight the clinical relevance of MRV infections and support further investigation of their epidemiology. In contrast to rotaviruses, which are classified in the family Sedoreoviridae within the order Reovirales and cause the annual deaths of 200,000 children in developing countries [41], MRVs exhibit lower pathogenicity but remain capable of significant genetic variation through reassortment and recombination events [42].

The detection of MRV1_SP/BR in an open stream running through a densely populated favela documents its presence in a sewage-impacted urban environment. Environmental detection, sequence similarity, and evidence of zoonotic transmission should be clearly distinguished. Although MRV1_SP/BR shares high nucleotide identity with sequences previously detected in human and animal hosts, these similarities alone do not establish the host origin of its genome segments or demonstrate cross-species transmission. Isolation in cell culture documents the presence of infectious MRV in the sampled water, but does not establish infection in exposed humans or animals. No human or animal clinical samples were analyzed in this study, precluding direct comparison with locally circulating clinical strains and assessment of epidemiological links. Future surveillance integrating environmental sampling with human clinical diagnostics and sampling of local animal populations could help investigate potential exposure pathways and transmission links.

Notably, none of the bat species linked to MRV segments are known to occur in São Paulo city. A possible explanation for this is the limited representation of MRV sequences in databases, particularly from rodents and humans, complicating host assignment. As more genomes become available, segments currently associated with bats may cluster with local bat, rodent or human strains [26,43,44]. Furthermore, only samples collected in 2020 were available for this study, meaning the current prevalence of MRV in São Remo remains unclear. Finally, because a neutralization assay was not performed, the MRV-1 designation used here represents an S1-based genetic classification rather than antigenic confirmation; type-specific neutralization would be required to confirm its serotype.

These findings highlight the importance of expanding the MRV sequence database in Brazil. The combination of untreated wastewater and viruses from potentially diverse sources in settings such as São Remo highlights the importance of monitoring environmental viral diversity and reassortment. Environmental monitoring, particularly through stream sampling, can help characterize MRV diversity and guide further investigation of potential exposure pathways. While MRVs are not considered major human pathogens at present, they may serve as useful sentinels for fecal contamination and environmental viral diversity, highlighting the value of integrated surveillance for tracking viral evolution [11,16].

Our findings broaden the known orthoreovirus genomic diversity in Brazil by documenting a reassortant MRV strain in a sewage-impacted urban stream. The genomic characterization of MRV1_SP/BR contributes to understanding MRV evolution and provides a foundation for future studies integrating environmental, human, and animal surveillance.

## Figures and Tables

**Table 1:** Highest nucleotide identities for each gene segment of the Brazilian São Remo MRV1_SP/BR strain.

Segment	Genotype	Nucleotide Identity (%)	Common Name	Host/Source	Country	GenBank Accession Number	Year
L1	1	98.9	—	*Homo sapiens*	Switzerland	KX932029	2015
L2	2	98.3	Big Brown Bat	*Eptesicus fuscus*	USA	OP057398	2017
L3	2	98.9	Big Brown Bat	*Eptesicus fuscus*	USA	OP057399	2017
M1	2	98.3	Steppe whiskered bat	*Myotis aurascens*	Republic of Korea	OQ789610	2020
M2	3	98.5	Kuhl’s pipistrelle bat	*Pipistrellus kuhlii*	Italy	KU194662	2011
M3	2	98.2	Kuhl’s pipistrelle bat	*Pipistrellus kuhlii*	Italy	KU194671	2011
S1	1	97.4	—	Wastewater	Canada	OR074680	2012
S2	2	98.3	Kuhl’s pipistrelle bat	*Pipistrellus kuhlii*	Italy	KU194673	2011
S3	1	99.9	Short-nosed fruit bat	*Cynopterus sphinx*	China	KX263315	2016
S4	1	98.2	Common bent-wing bat	*Miniopterus schreibersii*	Republic of Korea	MW582631	2019

**Table 2:** Nucleotide identity between the Brazilian São Remo MRV1_SP/BR strain and the previously reported Brazilian MRV-3 strain TO-151/BR, detected in feces from a child with diarrhea in Tocantins, northern Brazil.

Segment	Nucleotide Identity (%)	GenBank Accession Number
L1	88.7%	MN022937
L2	84.8%	MN022938
L3	94.5%	MN022939
M1	92.8%	MN022934
M2	89.1%	MN022935
M3	89.7%	MN022936
S1	47.0%	MN022930
S2	85.4%	MN022931
S3	88.0%	MN022932
S4	92.0%	MN022933

## Data Availability

The complete genome sequences of MRV1_SP/BR are available in GenBank under accession numbers PV209793–PV209802.

## Supplementary Materials

The following supporting information can be downloaded at https://www.mdpi.com/article/10.3390/v18101161/s1, Supplementary Table S1: Segment-specific sequencing metrics for the MRV1_SP/BR genome. Figure S1: Phylogenetic tree of the complete L1, L2 and L3 segments of the Brazilian São Remo MRV1_SP/BR strain (indicated by black arrows). Different hosts/sources are highlighted in different colors; Figure S2: Phylogenetic tree of the complete M1, M2 and M3 segments of the Brazilian São Remo MRV1_SP/BR strain (indicated by black arrows). Different hosts/sources are highlighted in different colors; Figure S3: Phylogenetic tree of the complete S2, S3 and S4 segments of the Brazilian São Remo MRV1_SP/BR strain (indicated by black arrows). Different hosts/sources are highlighted in different colors.
