# Supplementary material for: First Identification and Genomic Characterization of Mammalian Orthoreovirus Type 1 in a Heavily Polluted Stream from an Underserved Urban Settlement in Brazil
Source: Viruses. 2026 Oct 19;18(10):1161. doi: 10.3390/v18101161 (PMC13709415; doi:10.3390/v18101161)
Supplement: Supplementary file 1 [file viruses-18-01161-s001.zip › viruses-4473757-supplementary.pdf]

Supplementary Material

Supplementary Table S1. Segment-specific sequencing metrics for the MRV1_SP/BR genome.

Segment	Consensus length (nt)	Mapped reads	Mean coverage (×)
L1	3,963	4,283	3,710
L2	3,870	4,090	1,712
L3	3,873	4,023	980
M1	2,259	2,669	1,100
M2	2,127	2,629	1,225
M3	2,166	2,936	450
S1	1,454	1,534	610
S2	1,271	2,081	3,232
S3	1,101	1,470	180
S4	1,295	1,405	652

Supplementary Material

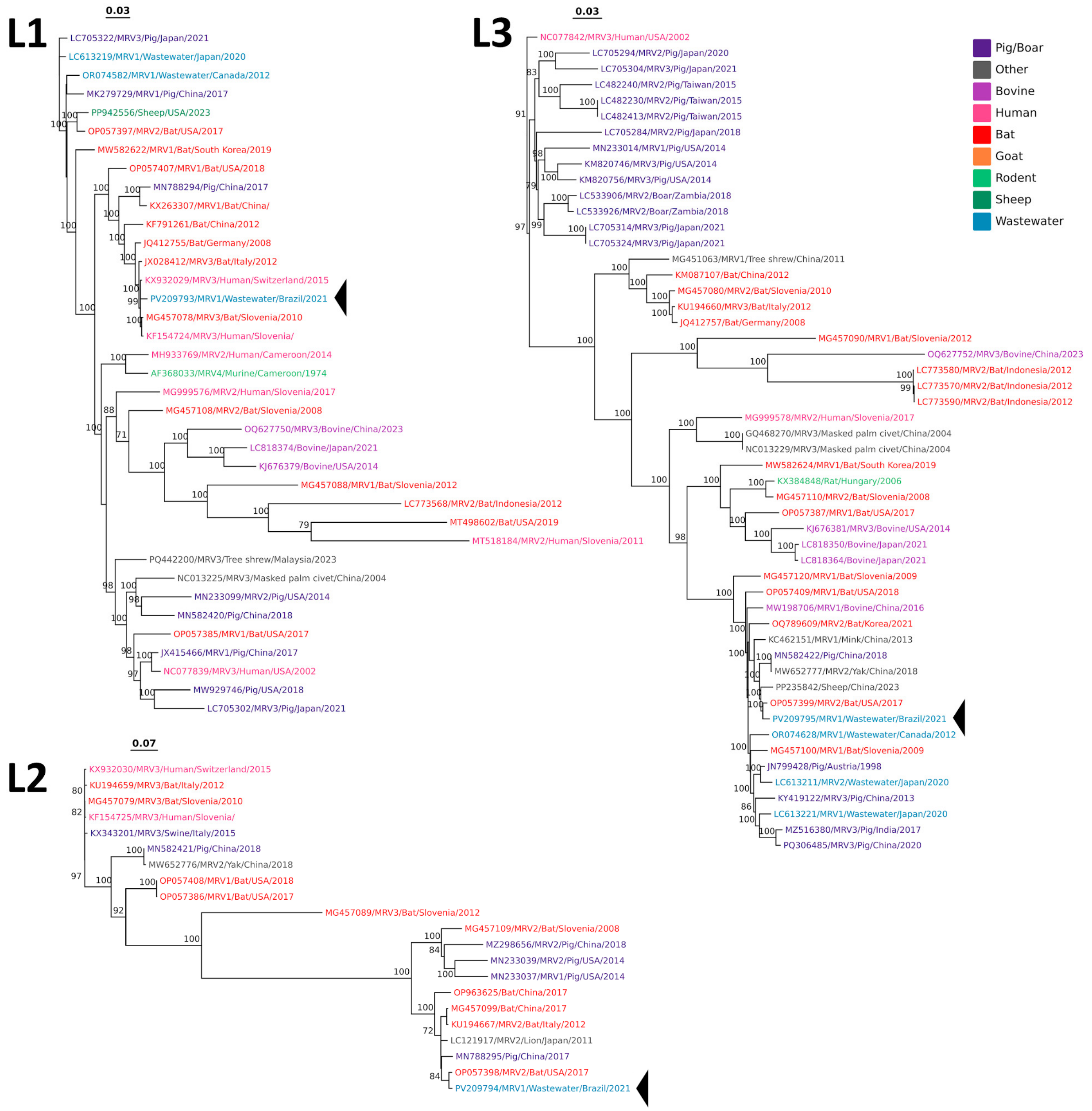

Figure S1. Phylogenetic tree of the complete L1, L2 and L3 segments of the Brazilian São Remo MRV1_SP/BR strain (indicated by black arrows). Different hosts/sources are highlighted in different colors.

Supplementary Material

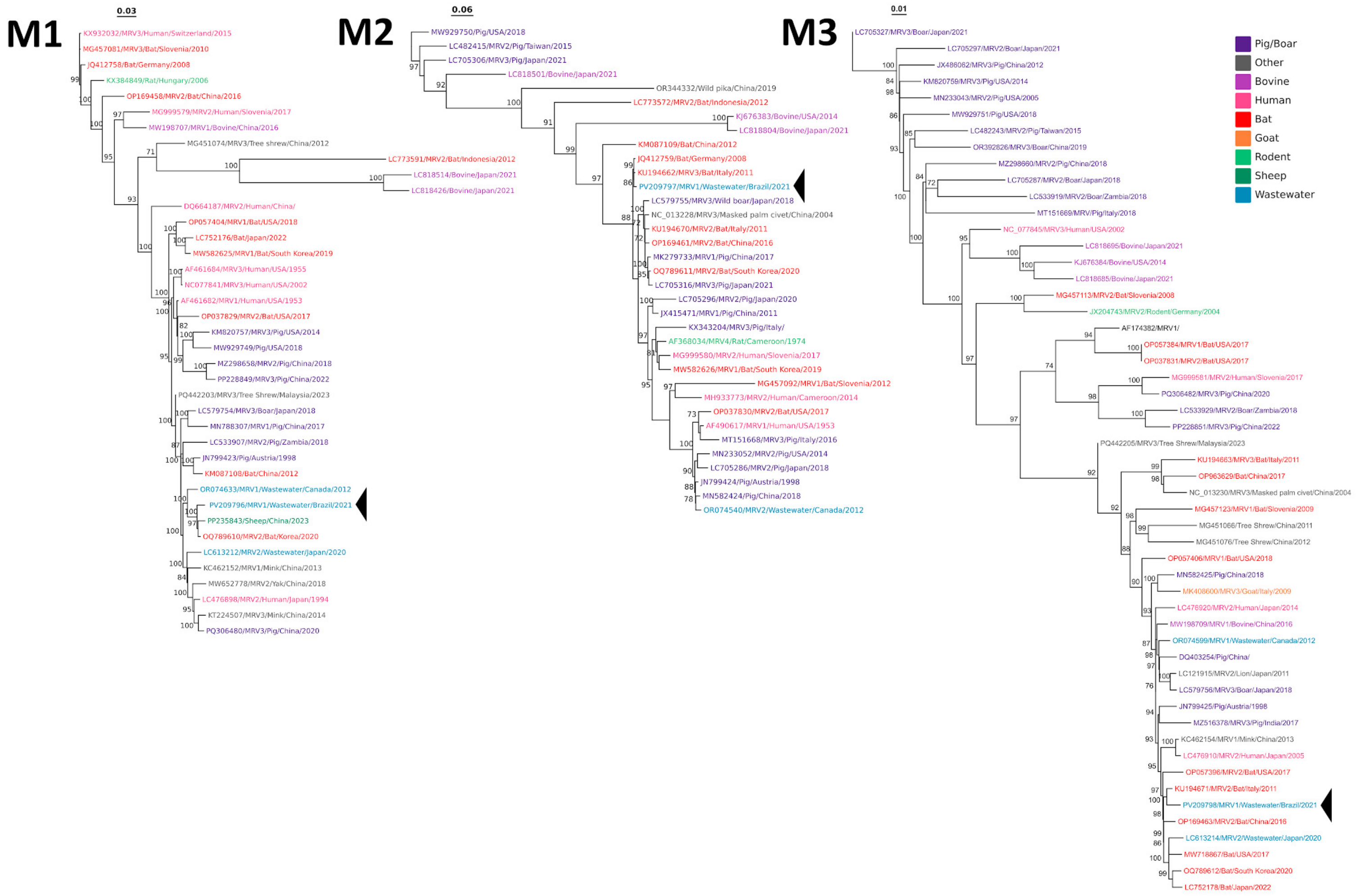

Figure S2. Phylogenetic tree of the complete M1, M2 and M3 segments of the Brazilian São Remo MRV1_SP/BR strain (indicated by black arrows). Different hosts/sources are highlighted in different colors.

Supplementary Material

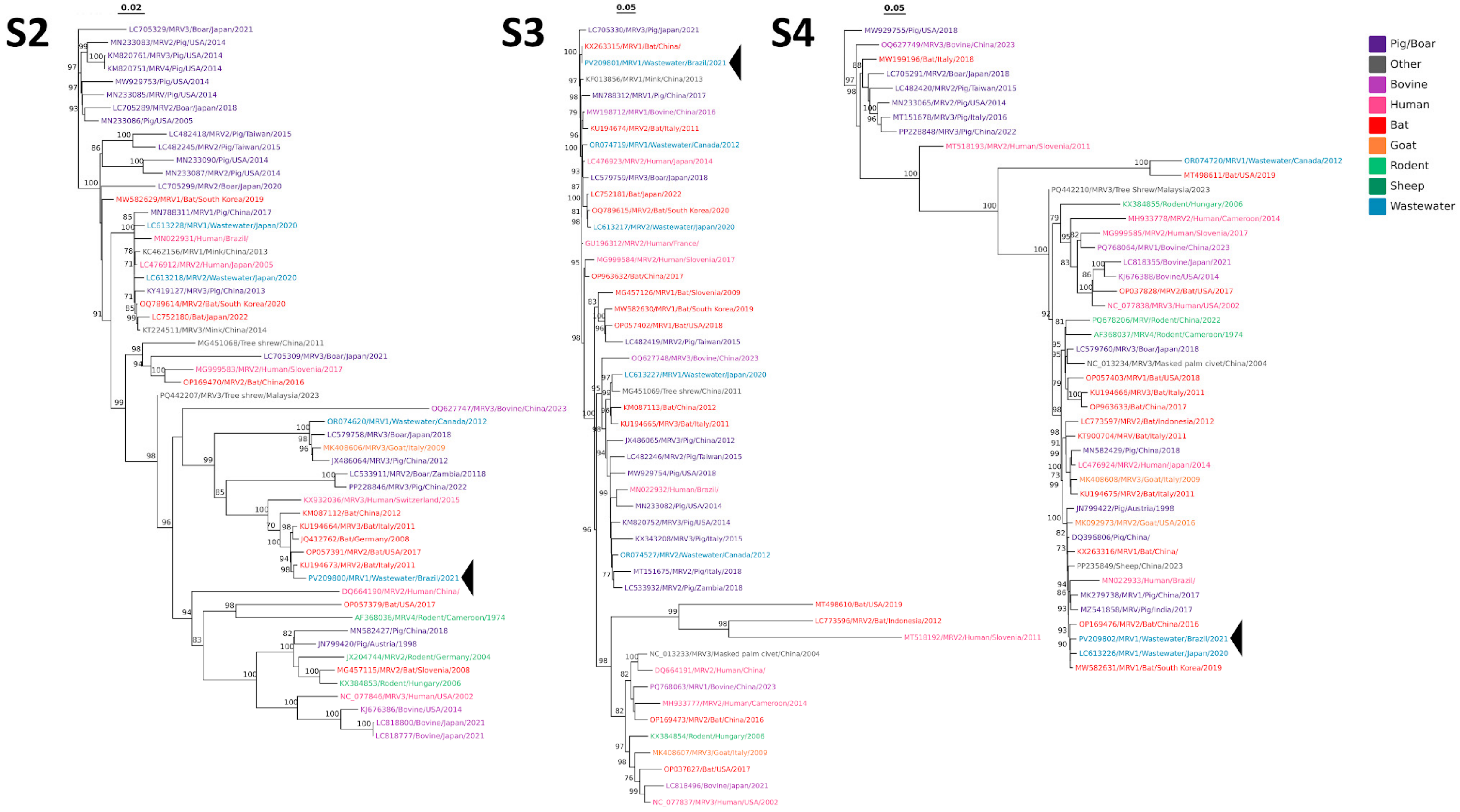

Figure S3. Phylogenetic tree of the complete S2, S3 and S4 segments of the Brazilian São Remo MRV1_SP/BR strain (indicated by black arrows). Different hosts/sources are highlighted in different colors.
